# USING HEALTH INFORMATION EXCHANGE DATA FOR SOCIAL RESEARCH: CHALLENGES AND OPPORTUNITIES

**DOI:** 10.1093/geroni/igad104.3688

**Published:** 2023-12-21

**Authors:** Allen Glicksman, Ashley Ritter, Steven Albert, Adam Davey, Ari Friedman, Lauren Ring, Misha Rodriguez, Michael Liebman

**Affiliations:** NewCourtland, Philadelphia, Pennsylvania, United States; NewCourtland, Philadelphia, Pennsylvania, United States; University of Pittsburgh, Pittsburgh, Pennsylvania, United States; University of Delaware, Newark, Delaware, United States; University of Pennsylvania, Philadelphia, Pennsylvania, United States; NewCourtland, Philadelphia, Pennsylvania, United States; NewCourtland, Philadelphia, Pennsylvania, United States; IPQ Analytics. LLC, Kennett Square, Pennsylvania, United States

## Abstract

Data collected in encounters between health providers and older adults can provide an important source of information regarding the experience of older persons with limited English Proficiency (LEP) in the health care system further enhancing our knowledge about health access in disadvantaged populations. Using data acquired from the local health information exchange (HIE) our team examined usage patterns of health services by Spanish and Chinese speaking older adults in Philadelphia. The data, which became available in July, represents one of the first opportunities to use HIE data to study these issues. HIE data link EMR records from providers across a defined geographic area to deliver information on patients served through participating organizations. The initial sample included reports on almost 200,000 persons with 7.7 million encounters. Each record contains information on type of encounter, diagnoses, insurance status, and related items. While availability of these data opens the opportunity to explore a variety of questions regarding health service use, methodological challenges to using HIE data are significant. These include (but are not limited to): 1) different coding schemes for key variables (e.g., encounter type, diagnosis); 2) LEP status, geography, and coding schemes may be confounded; 3) coding schemes used by providers may change over time so longitudinal data can include multiple coding types for the same patient; and 4) significant variation in coding race/ethnicity may be introduced for the same patient. We present examples of such challenges and approaches for resolution to enable and facilitate our analyses.

